# Population health in primary care

**DOI:** 10.3389/fmed.2024.1369741

**Published:** 2024-03-14

**Authors:** David A. Haggstrom, Robert A. Gabbay, William L. Miller, Jenna Howard, Benjamin F. Crabtree

**Affiliations:** ^1^VA Health Services Research & Development Center for Health Information and Communication, Roudebush VAMC, Indianapolis, IN, United States; ^2^Division of General Internal Medicine and Geriatrics, Department of Medicine, Indiana University School of Medicine, Indianapolis, IN, United States; ^3^IU Center for Health Services Research, Regenstrief Institute Inc., Indianapolis, IN, United States; ^4^Harvard Medical School, Boston, MA, United States; ^5^American Diabetes Association, Arlington, VA, United States; ^6^Department of Family Medicine, University of South Florida Morsani College of Medicine Lehigh Valley Health Network, Allentown, PA, United States; ^7^Department of Family Medicine and Community Health, Division of Research, Rutgers Robert Wood Johnson Medical School, New Brunswick, NJ, United States

**Keywords:** primary care, population health, patient centered care, community health services, health information technology (health IT)

## Abstract

Population health in the United States continues to lag behind other wealthy nations. Primary care has the promise of enhancing population health; however, the implementation of a population health approach within primary care deserves further consideration. Clinicians and staff from a national sample of 10 innovative primary care practices participated in a working conference to reflect upon population health approaches in primary care. A series of small- and large-group discussions were recorded, transcribed, and coded through an immersion/crystallization approach. Two prominent themes emerged: (1) Transitioning to a population health focus generally develops through stages, with early implementation focusing on risk stratification and later, more advanced stages focusing on community health; and (2) Several inherent barriers confront implementation of a population health approach, including tensions with patient-centered care, and limitations of health information technology. A broader conceptualization of population health in terms of community health could more effectively allow partnerships among primary care, large health care systems, public health organizations, patients, and other partners in the community.

## 1 Introduction

Countries and populations with adequate access to high-quality primary care experience reduced all-cause mortality (1), better preventive care (2), and reduced health disparities (3). For individuals to accrue long-term benefits of improved health from primary care, there needs to be enhanced overall population health, an issue that came into greater relief during the COVID pandemic. The preventive and community emphases of primary care make it a focal point for changes in health care system orientation toward a population health perspective.

After the COVID pandemic, what is meant by population health is increasingly interrogated within the health care setting. Initial attempts to address population health were driven in part by the Institute for Healthcare Improvement’s (IHI) promulgation of the Triple Aim, which calls for improvements in patient experience, population health, and *per capita* cost (4). Operationalizing a population focus in a meaningful way, without further weakening primary care, remains an open challenge (5).

Given the national, collective interest in promoting population health approaches in primary care, we sought to learn from primary care practices that have already adopted a population-based orientation. To this end, proceedings from a working conference of representatives from innovative primary care practices that was previously convened were analyzed to learn about their experiences, successes, and barriers. The findings highlight issues and questions to consider now as the U.S. healthcare system increasingly takes on the goal of developing a population focus in health care delivery.

## 2 Methods

### 2.1 Participant selection

According to the American Academy of Family Physicians, a primary care practice can be defined as a set of healthcare professionals who serve as a patient’s entry point into the healthcare system, provides patients with ready access to their own personal physician and healthcare team, and is generally located in the community served. For this study, participating practices were selected from a Robert Wood Johnson Foundation list of workforce innovators in primary care. This list was the product of a literature search using 40 key terms to identify peer-reviewed literature about primary care workforce innovations in the United States since 2001 (6, 7). More than 4,000 articles from the initial search were screened, yielding 331 relevant articles. Authors of these articles were contacted and asked to nominate innovative practices, using a snowball sampling strategy. This process led to the identification of 151 individual practices, each of which was contacted by phone for an interview that was used to complete a two-page summary table with details for each practice, including the degree of innovation and sustainability (8). From this list, we identified 19 practices with a strong population health approach to primary care and/or team-based care. Blinded to practice identity, the steering committee ranked these, and the 10 highest-ranked practices were invited to participate in the conference. All but one practice agreed, so the next-ranked practice was invited. Participating practices chose one representative to attend the conference. The participants included five physicians, one registered nurse (with PhD), and four practice administrators. They represented practices from nine states (four each from the Midwest and East, and one each from the South and West), including four family medicine practices (three private, one health system owned), two internal medicine practices (one hospital owned, one system owned), one pediatrics practice (private), one nurse-led community clinic (co-owned by university and community), and two federally qualified health centers. Conference participants also included two nationally recognized experts in the topic areas (DH and RG), four dissemination consultants, and three additional research team members (BFC, WLM, and JH). Finally, four patient community representatives participated during the first day of the conference.

### 2.2 Conference organization

The working conference was held in Denver, Colorado in March 2013 with support from the Agency for Healthcare Research and Quality (AHRQ). During the course of the two-and-a-half days, conference organizers aimed to create an environment for clinicians and staff from ten innovative primary care practices to reflect with system change experts and content experts on strategies for implementing two elements of primary care: (1) population-based care and (2) team-based care. These topics were discussed in a series of large and small break-out group formats, with the aim of facilitating maximum interaction.

### 2.3 Data collection

Notes were taken by trained observers and all sessions were digitally recorded (20 h total). Additionally, practice representatives wrote a short summary (2–4 pages) of their practices’ innovations before the conference and then participated in a formal, one-on-one interview with one of three qualitative researchers (including JH) while at the conference (45–60 min).

### 2.4 Data analysis

An immersion/crystallization approach to analysis was used, whereby the team iteratively engaged with the raw data to identify emergent themes and insights. Initially, three of the authors (DH, RG, BC) reviewed the conference data to identify broad thematic areas. Through a series of conference calls with the larger research team and multiple re-readings of the data, these themes were refined. Another sub-group of authors (RG and JH) then did an intensive re-reading to corroborate the team’s interpretations and check for consistency. Finally, one author (WLM) went back through the data to seek alternative interpretations with which to challenge the group and further refine nuances of the interpretation.

## 3 Results

A pair of prominent themes emerged through the conference deliberations: First, *transitioning to a population health focus may develop through stages.* Initial discussions conceptualized population health primarily in terms of risk stratification (early stage), which participants recognized to be “low-hanging fruit.” As the dialogue progressed, a more comprehensive view of population health emerged that framed the optimal approach in terms of community health (advanced stage). This conceptualization emerged as the group’s preferred view of population health. Throughout both phases of discussion, a second theme was prominent: *Several inherent barriers confront implementation of a population health approach.* These include the tension between population health and patient-centered care, and the limitations of health information technology.

### 3.1 Transitioning to a population focus may develop through stages

#### 3.1.1 Early stage: risk stratification

Participants expressed that the purpose for risk-stratifying patient panels is to develop clinic interventions for different groups, with varying healthcare needs. Members of the health care team should have access to the registries of these groups for population-directed care (e.g., physicians for prioritization, nurse care managers for outreach, etc.). Participants agreed that team-based care must be in place before population management can be successfully pursued.

Participants questioned the extent to which practices are thinking about populations outside of the health conditions typically represented by incentivized quality metrics. For example, cancer survivors were highlighted as an emerging population, and none of the practices had any experience handling this population differently than other patients. Behavioral risk factors were considered an important factor to leverage in identifying populations for outreach and intervention. One physician reflected on this possibility:

How do we begin to screen our populations not only for high-risk disease, but high-risk behavior, or patient behavior? You may [eventually] be able to screen very easily with our systems and tools [used] now for high complexity disease.

Existing enterprise risk stratification tools, however, were noted to be limited in that they typically depend on clinical parameters, with limited ability to include patient behavior and social determinants in risk assessment.

#### 3.1.2 Advanced stage: responsibility to the local community

Beyond risk stratification, the participants developed a vision for what population health could be. The ideal that emerged revolved around considering the local community to be the locus of population health. One practice in particular had begun to actualize this ideal and shared several different approaches, such as building relational communities through community-based participatory approaches:

What we did was intentionally put our [clinic sites] back into community-based organizations. So, we operate out of two trusted organizations…where we are surrounded by everything from gymnasiums to childcare… So, when I need to make a referral…we do the ‘out-grab’ phenomena and we take people by the ear and walk them down to the GED program, because probably what’s going to make the difference to them and their family is getting…a GED and moving up the ladder.

This practice also forwarded the notion that population health could be considered as “concentric circles” of need, with the primary care practice in the center but located and grounded within the surrounding community.

Innovative outreach activities described by other participants included health education in schools or libraries, support for local natural disaster victims, and home visits. Furthermore, practices can create communities within themselves through lay patient advisors, group visits, or virtual support networks via social media. Other specific examples included a pregnancy group among Latinx women and support for a local soccer team from among the patients’ community.

Participants agreed that the paradigm shift from clinic-based to community-based population health is even larger than the shift from autonomous, individual practitioners to team-based care. Such a shift represents a fundamental change in the relationship between physician and patient by moving it to the multiple relationships within a population or community. As one academic participant summarized:

We’re speaking now of changing that covenant (between patient and physician) to potentially be one about life together, which is not about life as autonomous individuals. But it’s life about taking mutual responsibility for each other with our different sets of skills, with a mutual respect that preserves the agency that each of us brings into our life.

### 3.2 Several inherent barriers confront implementation of a population health approach

Throughout the discussion about how population health approaches develop through stages, and an emerging vision of the potential for community health, participants also highlighted two important barriers that work against the implementation of a population health approach.

#### 3.2.1 Tension between population health and patient-centered care

One barrier to developing a population health approach raised throughout group discussions was the tension between “population health” and “population management.” Participants reflected upon the implications of carving up patients into different sub-populations, cautioning about the potential for this approach to serve as a means to simply measure, track, and control clinician behavior, rather than serve clinical value. The management of quality metrics was critiqued as potentially undermining the clinical goals of competent, caring clinicians. One physician participant highlighted the point:

The more healthcare and primary care is seen as a problem to be solved, the more the usual ways of solving that…are going to happen… ‘Let us partition everything, and let us measure; let us get metrics. And by God, we cannot count on the people on the frontlines to be doing the right thing. So, let us hold their toes to the fire for every little metric’.

The group discussed the need to move beyond disease management toward being more patient-centered, even with a population focus. By creating incentives to reward specific physician behaviors, population management (defined in terms of performance measures), may not lend itself to a patient-centered focus on an individual’s care. As one physician participant noted: *The patient’s goals are not inevitably the physician’s or insurance company’s goals*. Participants agreed that metrics may conflict with the individual patient’s self-perceived need or preference, and even that of the clinician.

#### 3.2.2 Limitations of health information technology

The second barrier that surfaced regularly among participants was the limitations of traditional health information technology (HIT) to facilitate population health. Participants’ experiences highlighted that data is not usually shared, even between organizations using the same EHR system. It was also noted that since medical care itself is rapidly evolving and standards of care often change, current systems can be out-of-date before they are even implemented. One participant commented that health information systems need to anticipate the future: *We need systems that evolve with care, not just reporting mechanisms*.

Participants suggested that in order for the EHR to be supportive of population health, clinician input on EHR development is critical. One family medicine participant made a case for clinicians to be *at the table* with the HIT developers because non-clinician programmers *are unlikely to have a clue about client flow and healthcare delivery*. This same practice reported a relationship with a HIT development company and described sitting *arm in arm with programmers* while they created the system: *Doing the clinician review before we say “okay” made it a different kind of product*.

Finally, the role of HIT was discussed in relation to the group’s emerging ideal of population health as grounded in community health. One practice had targeted the measurement of social determinants of community health in partnership with their academic nursing partner:

We do annual community assessments. We send a team out, and they come up with some pretty wonderful stuff. And one of the things they came up with was that the hospitalization rate for asthma in this particular community that we are serving…was four times what it was elsewhere in [the city]…

As this nurse practitioner explained, their practice was able to use this collected data to *engage a very wide group of partners* … *[including] the neighborhood center that is our host partner and the Housing Authority…as the Housing Authority prepares to rebuild the 726 units that we live in the midst of, they have taken an entire block and turned them into healthy homes*.

There was wide recognition that further study of how HIT can facilitate collecting, aggregating and using such data would be a critical avenue for future research.

## 4 Discussion

Several messages regarding how population health can be addressed in the context of primary care were generated from the conference dialogue. One key principle was that the implementation of a population health approach occurs in progressive stages. In the early stage, practitioners see the development of disease registries as foundational. While participants unanimously endorsed the value of such registries, caution was raised that these tools should not be treated as an end point or equated with population health care itself. Disease registries should be considered the initial, feasible, “low-hanging” fruit in a more extended developmental process.

There was resistance to defining and measuring population health solely in terms of traditional HEDIS or MACRA-type quality or performance measures. As a practical matter, disease registries were noted to focus on a single disease, not reflecting the clinical reality that many patients have several chronic diseases and therefore live in multiple registries. Thus, subsequent outreach runs the risk of being confusing, duplicative, or poorly coordinated. Other surveys have also found primary care clinicians to be negative about the reliance on quality metrics (9). Another reason for antipathy toward quality metrics was their implicit displacement of clinician judgment, especially with regards to what level of patient comorbidity justifiably mitigates the benefit of recommended clinical interventions. Traditional quality measures also tend to segment the population without considering more universal therapeutic goals such as patient-centered decision-making. One consequence is that strong provider advocacy of recommended clinical interventions may adversely affect the patient-provider relationship (10). Others have found that shared decision-making may better facilitate the coordination of goals between providers and patients (11). Tools to measure the quality of decisions have been considered, but are not widely deployed (12, 13).

**Figure 1:**
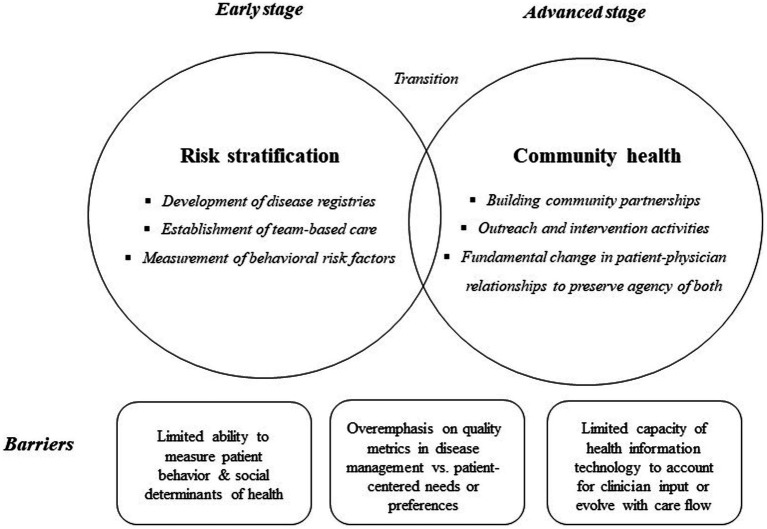
Transition from early to advanced stage primary care practice and barriers.

Ideally, the goals of a population health approach are much more extensive than what a registry alone can facilitate. During the course of dialogue, participants began to envision a next stage in developing a population health approach, in which the incentives change from being driven by quality measures and patient experience within the healthcare clinic’s panel to a focus upon the *health of the population in the community served* by primary care practices. What innovators proposed was a co-created vision, while cautioning about challenges that may stand as barriers to achieving the goals of community health. The primary care field is now faced with the task of developing models of advanced care delivery that can systematically overcome barriers to change, including limited abilities to measure behaviors and social determinants of health, an overemphasis on quality metrics, and limited capacity of health information technology (Figure 1). Similar to how national organizations (e.g., AAFP, National Committee for Quality Assurance, etc.) developed criteria for Patient-Centered Medical Home (PCMH) attributes, the same type of careful articulation will need to occur for advanced population health approaches. Demonstration projects are needed to test and refine new models, similar to the previous development of the PCMH model (14–16).

Complexity science provides a framework through which to view the progressive stages in the development of population health approaches in primary care (17). Key principles of a complex adaptive system (CAS) that are useful for understanding primary care practices and their communities include (i) a CAS consists of “agents” (e.g., physicians, nurses, and office staff) with the ability to learn and freedom to act in improvisational ways; (ii) while the agents are often individuals, they may also be teams (within practices), organizational processes (performance measurement), and technical capacities (information technology); (iii) agents are connected in nonlinear ways such that one agent’s actions changes the context for other agents (within practices and practices embedded in communities); and (iv) the quality of the interactions among agents (individuals, practices, and communities) is more significant than the quality of the agents alone.

Our findings have several limitations. First, the conference occurred a decade ago which means that temporal changes in healthcare delivery may limit the applicability of our results; however, the last major legislative healthcare reform (the Affordable Care Act) was enacted in 2010, and the results herein still appear very relevant to the health policy landscape today. While the data derive from discussions and interviews with 10 representative primary care practices, widespread generalizability may be limited. While this finite practice number was essential to facilitate engagement and the depth of deliberation, the scope of experience reflected in the data may be narrower. Yet generalizability was not the primary purpose of this study; rather, the intent was to focus upon high performers or innovators. Several different practice types were also included (i.e., family medicine, internal medicine, pediatrics, federally qualified health centers, and a nurse-led community clinic); however, the sample again was not large enough that any differences in the types of innovations were observed across practice types. An additional methodologic limitation is that a significant portion of the information shared by participants occurred in the context of group discussions. This design was chosen to facilitate a richer, more interactive exploration of the topics. However, it is possible that some amount of group think can occur (18), which would inadvertently minimize less dominant perspectives.

Understanding of barriers and facilitators to population health, as well as its long- and short-term goals, is still critically needed within the context of the U.S. health care system. Previously, the understanding of population health in the context of primary care has largely been operationalized as the health of the patient panels served directly by a practice, usually driven by reimbursement incentives around particular quality metrics. This constrained panel-based definition of “population health” is perhaps better named “population management,” and limits the ability of primary care to address the social determinants that influence the health of the larger population where practices are located (Table 1). A more expansive and transformative understanding of population health includes all people living in the geographic area or community served by a primary care practice, a definition whose feasibility and merits have been previously discussed in the context of accountable care organizations (19).

**Table 1:** Summary of differences between population management and population health.

Domain	Population management	Population health
Primary driver	Quality metrics	Unique health needs of the local community
Intervention focus	Exclusively medical	Includes social determinants of health
Population	Practice patient panel	Local community, whether or not patients visit the clinic

Performance evaluation designs tend to assess the quality, cost, and outcomes associated with the implementation of care processes, but not how the model impacts the health and vibrancy of the broader communities in which patients live. One of the fundamental attributes of primary care is *sustained partnership and personal relationships over time with patients known in the context of family and community* (20). A broader frame for population health, defined in terms of community health, more effectively allows partnerships among large health care systems, primary care, public health organizations, patients, and other partners in the community.

## Data availability statement

The datasets presented in this article are not readily available due to the nature of the research and human subjects protections. Further requests should be directed to the corresponding author.

## Ethics statement

The studies involving humans were approved by Institutional Review Board at Rutgers Robert Wood Johnson Medicine School. The studies were conducted in accordance with the local legislation and institutional requirements. The ethics committee/institutional review board waived the requirement of written informed consent for participation from the participants or the participants’ legal guardians/next of kin because the research presented no more than minimal risk of harm to subjects.

## Author contributions

DH: Conceptualization, Data curation, Formal analysis, Investigation, Methodology, Resources, Supervision, Visualization, Writing – original draft, Writing – review & editing. RG: Data curation, Formal analysis, Methodology, Supervision, Validation, Writing – original draft, Writing – review & editing. WM: Data curation, Formal analysis, Methodology, Supervision, Validation, Writing – original draft, Writing – review & editing. JH: Data curation, Investigation, Methodology, Project administration, Writing – original draft, Writing – review & editing. BC: Conceptualization, Data curation, Formal analysis, Funding acquisition, Investigation, Methodology, Project administration, Resources, Supervision, Validation, Visualization, Writing – original draft, Writing – review & editing.
